# Trawling bats exploit an echo-acoustic ground effect

**DOI:** 10.3389/fphys.2013.00065

**Published:** 2013-04-05

**Authors:** Sandor Zsebok, Ferdinand Kroll, Melina Heinrich, Daria Genzel, Björn M. Siemers, Lutz Wiegrebe

**Affiliations:** ^1^Sensory Ecology Group, Max Planck Institute for OrnithologySeewiesen, Germany; ^2^MTA–ELTE–MTM Ecology Research GroupBudapest, Hungary; ^3^Division of Neurobiology, Department Biology II, Ludwig-Maximilians-University MunichPlanegg-Martinsried, Germany; ^4^Graduate School of Systemic Neurosciences, Ludwig-Maximilians-University MunichPlanegg-Martinsried, Germany

**Keywords:** *Myotis daubentonii*, echo-acoustic mirrors, target detection, target discrimination, echo enhancement, trawling bats, ground effect

## Abstract

A water surface acts not only as an optic mirror but also as an acoustic mirror. Echolocation calls emitted by bats at low heights above water are reflected away from the bat, and hence the background clutter is reduced. Moreover, targets on the surface create an enhanced echo. Here, we formally quantified the effect of the surface and target height on both target detection and -discrimination in a combined laboratory and field approach with *Myotis daubentonii*. In a two-alternative, forced-choice paradigm, the bats had to detect a mealworm and discriminate it from an inedible dummy (20 mm PVC disc). Psychophysical performance was measured as a function of height above either smooth surfaces (water or PVC) or above a clutter surface (artificial grass). At low heights above the clutter surface (10, 20, or 35 cm), the bats' detection performance was worse than above a smooth surface. At a height of 50 cm, the surface structure had no influence on target detection. Above the clutter surface, also target discrimination was significantly impaired with decreasing target height. A detailed analysis of the bats' echolocation calls during target approach shows that above the clutter surface, the bats produce calls with significantly higher peak frequency. Flight-path reconstruction revealed that the bats attacked an target from below over water but from above over a clutter surface. These results are consistent with the hypothesis that trawling bats exploit an echo-acoustic ground effect, in terms of a spatio-temporal integration of direct reflections with indirect reflections from the water surface, to optimize prey detection and -discrimination not only for prey on the water but also for some range above.

## Introduction

In course of evolution, bats, as the only airborne mammals, adapted to a large variety of habitats. The species of this ecologically highly diverse group provide many morphological, physiological as well as behavioral adaptations e.g., of their sensory-motor system (Schnitzler and Kalko, 2001). Echolocating bats emit ultrasonic sounds and listen to the returning echoes reflected by objects in the environment. This enables bats to orient and hunt in complete darkness allowing prey detection, localization, and identification. But the biosonar system is prone to interferences. When bats use echolocation e.g., during foraging they have to deal with sound attenuation and masking effects. Amongst others, attenuation can be caused by atmospheric absorption losses that especially have a strong impact on high frequencies as they are used by bats (Lawrence and Simmons, 1982). Items close to the object of interest can create masking effects that impede prey detection (Fenton, 1990; Suemer et al., 2009; Bates et al., 2011). This so-called clutter interference can appear e.g., when hunting close to the ground or foliage. Hence, bats are not only morphologically adapted to their habitats (e.g., by wing shape) (Norberg and Rayner, 1987), but also by their echolocation signals (Schnitzler and Kalko, 2001; Siemers and Schnitzler, 2004; Wund, 2005). The differences in the echolocation call parameters (e.g., frequency, call duration, call intensity) are species-specific and also habitat-dependent. For example in vespertilionid bats, species that hunt in free airspace emit loud, narrowband echolocation calls to detect prey from a larger distance, whereas species that hunt near vegetation emit broadband echolocation calls to catch prey objects that are only a few centimeters in front of a clutter producing background (Schnitzler et al., 2003). Additionally the structure of echolocation signals can also differ with the behavioral task. In insectivorous bats for example, the echolocation signals during search, approach, and final buzz phase are very different (Schnitzler and Kalko, 2001).

One particularly interesting group consists of bats hunting almost exclusively above water surfaces. These so-called “trawling bats” hunt at low heights above water and capture fish or insects directly from or close to the surface. Water bodies like lakes, ponds, or streams are favorable hunting habitats for bats as the high abundance of insects provides a profitable food source (Zahn and Maier, 1997; Warren et al., 2000; Ciechanowski, 2002). In previous studies it was found that bats of this ecotype prefer to hunt over calm water compared to water e.g., covered by plants like duckweed, artificial objects, or turbulent, rippled water (Von Frenckell and Barclay, 1987; Mackey and Barclay, 1989; Boonman et al., 1998; Rydell et al., 1999; Siemers et al., 2001b; Siemers and Schnitzler, 2004). Two laboratory studies revealed that in the three European trawling-bat species' (*Myotis capaccinii*, *M. dasycneme*, and *M. daubentonii*) capture success was increased, compared to a clutter surface, when prey was presented on a smooth surface (linoleum screen) that mimicked the reflection characteristics of calm water. It was concluded that since the water surface acts as an acoustic mirror, echolocation calls emitted by bats are reflected away in acute angles from the bat. This creates an echo-image without, or just low clutter echoes and thus increases search efficiency as the prey echo is acoustically conspicuous (Siemers et al., 2001b, 2005). The search image for these bats was defined as “small and isolated echo-reflecting objects on or above an acoustically smooth surface” (Siemers et al., 2001a,b). This theory does not exclude inedible objects e.g., small leaves or debris on a water surface. However, one would expect efficient prey discrimination during flight to be beneficial to avoid catching inedible prey. But, in actively hunting bats no discrimination between edible and inedible objects that fit the general search image could be observed so far (Barclay and Brigham, 1994; Siemers et al., 2001b). Siemers et al. (2001b) showed that under semi-natural laboratory conditions trawling bats did not discriminate between a mealworm and a dummy presented on a linoleum screen.

The trawling bat *Myotis daubentonii* often hunts over rivers and streams (Jones and Rayner, 1988) providing a unidirectional water flow that often contains inedible objects as well as drifting prey. In a field study it was shown that *M. daubentonii* switches between trawling of prey from the water surface and aerial hawking (Todd and Waters, 2007), depending on the amount of clutter on the water surface.

Since previous studies were mainly designed to investigate prey detection on acoustic mirror and clutter surfaces without testing discrimination performance in detail, this study was designed to test prey detection *and* -discrimination. As the previous studies were conducted in the field, the participating animals behaved under natural conditions, but the participating number is an unknown factor. Whereas studies conducted in the laboratory allow control over the number of animals, but are limited in their imitation of natural surroundings. To benefit from both study types we formally quantify in the current study the effect of surface structure on both prey detection and -discrimination and on the echolocation behavior in a combined laboratory and field approach.

The main objectives of our study were to investigate the effect of the surface structure on the attacking and discrimination performance of the bats as well as flight path and the sonar vocalization features. These behavioral measures are discussed with respect to echo-acoustic features of the surface structures.

## Materials and methods

### Experimental animals

The species used in this study was the microchiropteran Daubenton's bat, *Myotis daubentonii*. It is found throughout Europe, foraging for insects above water surfaces using short (<5 ms), broadband frequency sweeps (95–25 kHz) for echolocation (Kalko and Schnitzler, 1989).

### Laboratory experiment

#### Animal housing

Laboratory experiments were conducted in July and August 2011 in the Max Planck Institute of Ornithology in Seewiesen, Germany. Data from five individuals of 12 h time shifted Daubenton's bats were recorded. The experiments were conducted under license of the responsible authorities and complied with German laws (LLUR 515/5327.74.1.6).

#### Experimental setup

**Figure 1:**
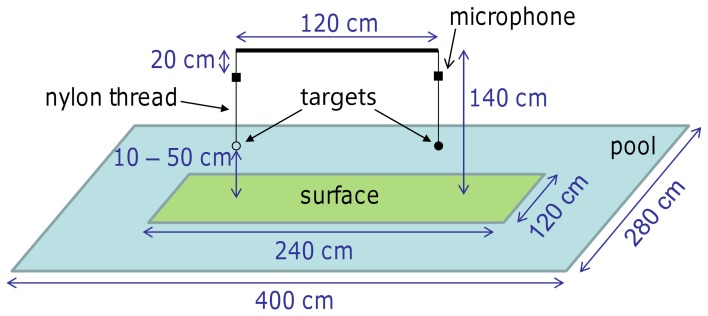
**Setup of the Field and Laboratory experiments.** In the two-alternative forced-choice paradigm the bat had the opportunity to attack one of the targets (mealworm or dummy). The surface beneath the targets was covered either with artificial grass or smooth PVC, or the place was left clear for the water surface. The two targets were always presented at the same height which was 10, 20, 35, or 50 cm above the surface. The horizontal bar holding the targets and microphones was attached to the ceiling of the Laboratory or, for the Field experiment, to a fishing rod anchored to the ground.

In the experiment a mealworm (larvae of *Tenebrio molitor*) and a dummy (1 mm black plastic disc with a diameter of 2 cm) were presented simultaneously. Both targets were hanging from easily exchangeable, variable-length nylon threads (Ø 0.15 mm) that were attached via small solenoids to a horizontal bar (Figure 1). The bar itself was suspended from the ceiling. This allowed an easy manipulation of the presented targets, e.g., target height (by variable lengths of nylon threads) and position (left or right side). The distance between the two targets was 1.2 m. The two targets were presented above either an artificial surface floating on the water or the water itself. The artificial surface measured 1.2 × 2.4 m. The targets were positioned such that each was hanging above the center of one half of a surface area with a minimum distance of 60 cm to the midline and the edges. The size of the experimental room was 3 × 7 × 3.5 m.

The experiment was monitored with synchronized normal- and high-speed video under infrared-light illumination and audio recordings. The normal-speed (25 frames/s) recordings were made by a single camera (WAT-902H2 Ultimate, Watec Co. LTD, Higashine, Japan) by means of the surveillance software (USB120 Server, Digiprotect, Frankfurt, Germany) to record the whole experimental process on the computer. The two high-speed digital video cameras (MV1-D1312I-160-CL-12, Photonfocus, Lachen, Switzerland; 100 frames/s, resolution 1312 by 1024 pixels, with specially developed software by Rauscher GmbH, Olching, Germany) recorded the last 5 s before a capture attempt. These high-speed recordings were used for reconstructing the flight path later on. The illumination was supported by two custom made stroboscopic flash lights (Department of Animal Physiology, University of Tübingen, Germany).

Acoustic signals were picked up by two ultrasound microphones (Knowles SPM0204, Itasca, IL, USA) that were attached 20 cm below the horizontal bar, i.e., vertically above the two targets. Echolocation calls were amplified and digitized with an Ultralite-mk3 (MOTU, Cambridge, UK) at a sampling rate of 192 kHz and recorded with Adobe Audition 2.0 (Adobe Systems Inc., San Jose, CA, USA) on the computer.

#### Experimental procedure

In the experimental conditions target height and surface type were varied. The surface types were defined as clutter surface (artificial grass matting with a height of 3 cm) or as smooth surface (water). Above the two different surfaces the targets were presented at four different heights (10, 20, 35, and 50 cm). This resulted in eight different experimental conditions which were presented following a pseudo-random protocol. The position (left or right) of the targets was also randomized.

Before each trial, both targets were hidden by two 70 cm high paper tubes while attaching them to the setup. This prevented bats from identifying and attacking the targets before trial start. In a trial, both targets were always presented simultaneously and at the same height. As the targets were suspended from nylon threads, they were not perfectly stationary, specifically, they often rotated slowly around their vertical axis.

### Field experiment

The Field experiment was conducted under license of the responsible authorities (Referat für Umwelt und Gesundheit, München, 641-304/P-12/7).

#### Recording sites and experimental setup

The field recording site was a shallow branch of the river Würm, located in Munich-Pasing, Germany (48° 8′ 0.59″ N/11° 26′ 52.37″ E, water depth: 10–20 cm). Data recording took place on 10 evenings between April and October 2011. The experiments were performed shortly after sunset when the first bats started hunting at the recording site. Depending on bat activity, recording sessions lasted about 3 h per night. To fit the requirements of the field research site, a slightly adapted version of the laboratory setup was used in the Field experiment (Figure 1). The horizontal bar holding the nylon threads with the targets was suspended from a fishing rod that was anchored to the ground. For video acquisition, a single high-speed digital video camera [Basler A602f, Ahrensburg, Germany, 95 frames/s with a Pentax H612A (TH) objective lens, Pentax Ricoh Imaging Co., Ltd., Tokyo, Japan] was used. The camera was positioned about 2 m from the targets and ~50 cm above the water surface. Red light illumination (two Philips IR PAR38E 150W, Amsterdam, Netherlands) was used to supply sufficient light for the camera. The microphones and their position were exactly the same as in the laboratory. Audio and video data were recorded in a 5 s ring buffer system implemented in MATLAB 7.5 (The MathWorks, Inc., Natick, MA, USA).

#### Experimental procedure

In the Field experiment, an additional, smooth surface type (PVC) was used with similar acoustic reflection properties as smooth water. The PVC board (1.2 × 2.4 m) was used as a control condition to rule out the possibility of potential performance changes of the bats being merely due to the artificial surface. Moreover, the water from the river was not smooth but, due to the irregular floor beneath the shallow, flowing water, the surface had small, regular waves, and ripples.

Unlike in the Laboratory experiment, only three different target heights were applied: 20, 35, and 50 cm. The presentation of these nine different conditions (three heights times three surfaces) followed a pseudo-random protocol where in successive nine trials each condition was presented once. Like in the laboratory, the position (left or right) of the targets was also randomized.

Before each trial, the bat species hunting at the setup were identified visually and acoustically by means of their echolocation calls with a Mini-3 Bat Detector (Ultra Sound Advice, London, UK). Later, this was verified by both video and sound analyses. Data analysis (see below) was the same as for the Laboratory experiment, except that the single camera did not allow flight-path reconstruction, and acoustic data from the field was not evaluated.

### Data analysis

#### Attacking performance

A trial began when a bat initiated an attack or when it had circled around one or both of the targets at least three times. An executed attack was registered when the bat performed a final buzz and touched one of the targets or the threads. Later, the audio and video recordings of each trial were analyzed to correct for any wrong observations during the trials.

The data from each individual obtained in the laboratory was summarized and the attacking performance was calculated as the ratio of the number of attacks (independent of whether it was the dummy or the mealworm) divided by the number of trials where a bat initiated a trial according to the above criteria. In the water surface conditions, the attacking performance was always 100% independently of the target height (see results below), therefore it needed no statistical evaluation. For the statistical evaluation of the performance in the grass surface conditions, a General Linear Mixed Model (GLMM) was fitted on the arcsine transformed attacking performance data (as independent variable) with factors height (fixed effect) and individual (random effect).

As for the field results, it was not possible to distinguish different individuals; therefore only one performance value was calculated in each condition. In the water and PVC surface conditions, the attacking performance was maximal (100%) independently of the height (no statistics needed). To evaluate the effect of the height in the grass surface condition a Fischer's exact test was applied. All the statistical computations in this study were conducted in Statistica 8.0 (Statsoft Inc., Tulsa, OK, USA) and in MATLAB.

#### Discrimination performance

To calculate the discrimination performance only those trials were used in which an attack had been executed. An attack toward the mealworm was defined as a correct decision, an attack toward the dummy as a wrong decision. The discrimination performance was calculated as the ratio of correct decisions divided by all attacks in each condition.

For the laboratory results a GLMM was built on the arcsine transformed discrimination performance data (as independent variable) with the factors target height (fixed effect) and individual (random effect). This was done for the water and the grass surface conditions separately. The data obtained at the 10 cm target height conditions was omitted, as only one individual once attacked the targets offered at this height in the grass surface condition.

For the field results, the height effect was tested with the Fischer's exact test for all three surface conditions on the performance data.

A binomial test was used to test whether the probability of the mealworm choice was above 50% chance level. This was done separately for the Laboratory and the Field experiment on the pooled data.

#### Flight path analysis

The high-speed video recordings of the Laboratory experiment were used to reconstruct the flight paths for the trials of the 35 cm target height conditions. The calculations were made using the freely available DLTdv3 program written in MATLAB (Hedrick, 2008). After the flight path reconstruction the median and the quartiles from the water and the grass surface condition were calculated. This was done separately for each frame relative to the capture moment for the graphical presentation. The average flight height for each path was calculated and a GLMM was applied to test the effect of the individuals (random factor) and the surface (fixed factor).

#### Call analysis

Calls were analyzed with a custom written MATLAB program based on a program provided by Holger Görlitz. Calls were first high-pass filtered at 20 kHz. The frequency spectrum was then obtained by computing a 1024-point FFT (fast Fourier transform) over a Hanning window. Before calculating the frequency parameters the spectrum was fitted with an 18th-order polynomial to smooth out the ripples caused by constructive and destructive interferences between a call and reflections from the water surface. These interferences create higher and lower magnitudes, respectively, which are smoothed out by the polynomial fit. There was a continuous, narrow-band disturbance from a power supply in the recordings. For this narrow frequency range, the measured spectral magnitude was replaced by a linear interpolation. From the fitted spectrum, peak frequency, bandwidth and the −20 dB lower and upper cut-off frequencies were calculated. Due to reflections from the water, the analysis of the temporal call parameters was impeded. Depending on the pulse intervals (PIs), calls were separated into either Approach (15 ms < PI < 30 ms) or Buzz I phase (6.5 ms ≤ PI ≤ 15 ms). Kalko and Schnitzler (1989) measured a PI of 55–65 ms at the beginning of the Approach phase and 12–8 ms at the end. Here we used a rather narrow window to categorize the approach calls to ensure non-Approach calls were excluded. Buzz II calls with a PI shorter than 6.5 ms were not analyzed as the decreasing amplitude of the calls, the water reflections and the short PI impeded the analysis. In the following, Buzz I is referred to as Buzz.

To test the significance of the difference in peak frequency between the water and grass condition we applied a GLMM taking the surface as fixed factor and the identity of the individuals as random factor for each height (20, 35, and 50 cm) and phase (Approach and Buzz) separately (altogether six tests). We excluded the data from the 10 cm target height condition from this analysis, as we had only one recording in which the target was attacked. We did not analyze the echolocation calls obtained in the field, as the analysis of the laboratory data showed a highly significant individual effect for peak frequency (due to the lacking identity of the recorded bats in the field).

#### Ensonification and impulse response analysis

To quantify the structural properties of the surfaces, the PVC, and the grass matting were ensonified to obtain their impulse responses (IR). The IR is the echo reflected from an object when the object is ensonified with an acoustic impulse (Dirac impulse) of theoretically infinite shortness and infinite amplitude (Weissenbacher and Wiegrebe, 2003). The IR was calculated by cross-correlating the recorded echo with the original signal in the time domain.

A disc of the respective material (PVC or grass) with a diameter of 30 cm was positioned at a distance of 90 cm to an ultrasonic speaker (Matsushita EAS 10 TH 800D, Osaka, Japan), and a ¼ inch ultrasonic microphone (Brüel & Kjær 4135 with 2671 preamplifier and 2610 measuring amplifier, Nærum, Denmark) which was attached coaxially at the speaker front. The discs were ensonified from 10 different angles between 90° (sound impinging perpendicularly on the disc) and 0° (sound propagating parallel to the disc) in 10° steps. To measure the IR, white noise with a cut-off frequency of 96 kHz was created in MATLAB, sent to the DA/AD converter (MOTU Ultralite-mk3; sampling frequency 192 kHz), amplified (Toellner Toe 7606, Herdecke, Germany), and played via the ultrasonic speaker for the duration of 40 s. Simultaneously the echo was recorded by the ultrasonic microphone. Spectrograms of the IRs were calculated using a 64-point FFT over a Hanning window and an overlap of 95%.

## Results

### Attacking performance

**Figure 2:**
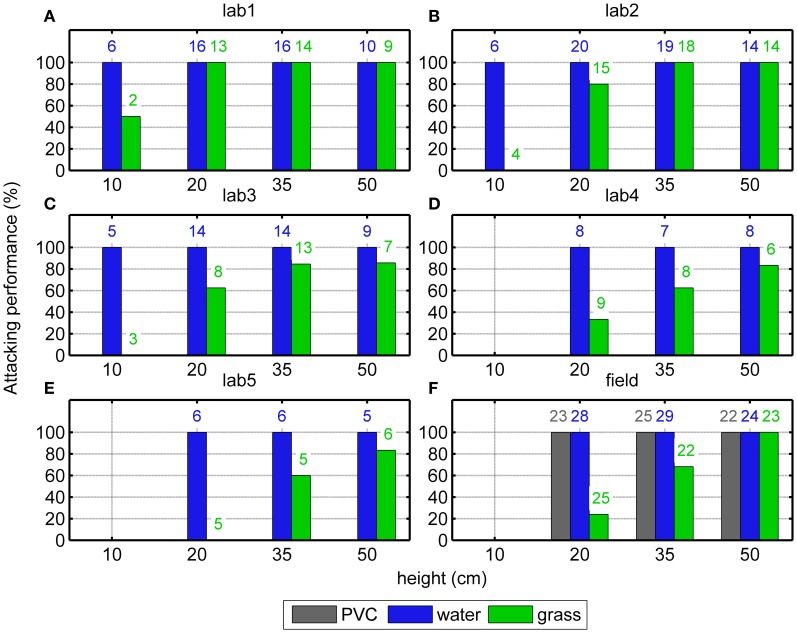
**Attacking performance above different surfaces at different heights.** The results from 5 bats in the laboratory (“lab1-5”, **A–E**) and from the field (”field”, **F**) show that the bats always attacked one of the targets when it was above water or PVC (blue and gray bars). In contrast, above grass (green bars), the bats' attacking performance drastically diminished with decreasing height. The numbers of the successful trials are shown on the top of the bars.

In the laboratory 347 trials were conducted with five individuals for eight conditions (four target heights, two surface types). For three individuals, data were obtained for four different target heights (10, 20, 35, and 50 cm). For two individuals, data were obtained for three different heights (20, 35, and 50 cm). After initiating a trial, all bats attacked one of the targets above water (Figures 2A–E, blue bars) independent of the target height. Above the grass surface, however, the performance deteriorated with decreasing target height (Figures 2A–E, green bars). The GLMM showed a significant effect of target height [*F*_(4, 10)_ = 20.0, *p* < 0.001] but also an effect of the individual [*F*_(3, 10)_ = 8.4, *p* = 0.003], meaning that the individual attacking performances above the grass surface differed significantly from each other.

In the Field experiment (218 trials) three different surfaces (PVC, water, or grass) and three different target heights (20, 35, or 50 cm) were presented. The same pattern of results as in the Laboratory experiment was observed: above water or PVC, the attacking performance was always 100% independently of target height (Figure 2F, blue and gray bars). However, above the grass surface, the attacking performance decreased monotonically with decreasing target height (green bars in Figure 2F, Fischer's exact test, *p* < 0.001).

### Discrimination performance

**Figure 3:**
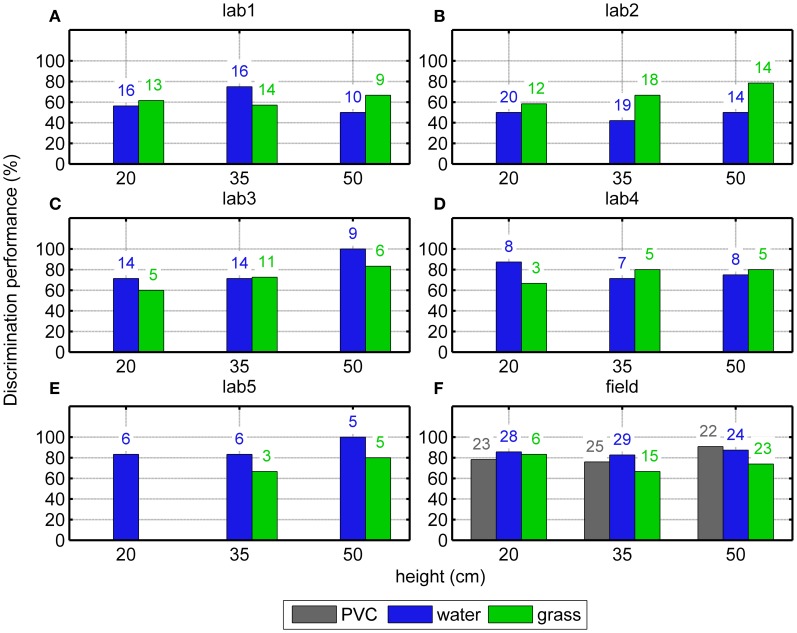
**Discrimination performance above different surfaces at different heights.** Results from 5 bats in the laboratory (“lab1-5”, **A–E**) and from the field (“field”, **F**) are shown. There is a statistically significant decrease of the discrimination performance in the laboratory animals over grass; however we have found no such significant relationship in the field. The number of the trials in which we observed an attack are shown on the top of the bars.

In the Laboratory experiment, data from six different conditions [three target heights (20, 35, or 50 cm) above two surface types (water or grass)] were used to evaluate the bats' discrimination of the mealworm from the disk dummy. In general, the bats attacked the mealworm more often than the dummy, regardless of height and surfaces. While the average discrimination performance across the five bats in the laboratory was only 66% correct (206 correct trials out of 313), this performance is statistically significant because of the high number of trials (One-sided Binomial Test, *p* < 0.001). The GLMM analysis shows no significant difference in the overall (height independent) discrimination performance between water and grass surfaces [GLMM, *F*_(1, 27)_ = 0.64, *p* = 0.43]. Also, discrimination performance did not deteriorate significantly with decreasing height of the targets above water [blue bars in Figures 3A–E, *F*_(2, 8)_ = 1.1; *p* = 0.37]. However, discrimination performance deteriorated significantly with decreasing height of the targets above the grass surface [green bars in Figures 3A–E, *F*_(2, 7)_ = 11.2; *p* = 0.007].

In the Field experiment, data from nine different conditions [three target heights (20, 35, or 50 cm) times three surface types (PVC, water, or grass)] were used. Similar to the Laboratory experiment, the bats attacked the mealworm significantly more often regardless of height and surface (One-sided Binomial Test, *p* < 0.001, Figure 3F). However, in none of the surface type conditions an effect of target height was found (Fischer's exact tests, *p* = 0.40 with PVC; *p* = 0.93 with water and *p* = 0.81 with grass). There was also no significant difference between the surfaces (Fischer's exact test, *p* = 0.075).

### Flight path analysis

**Figure 4:**
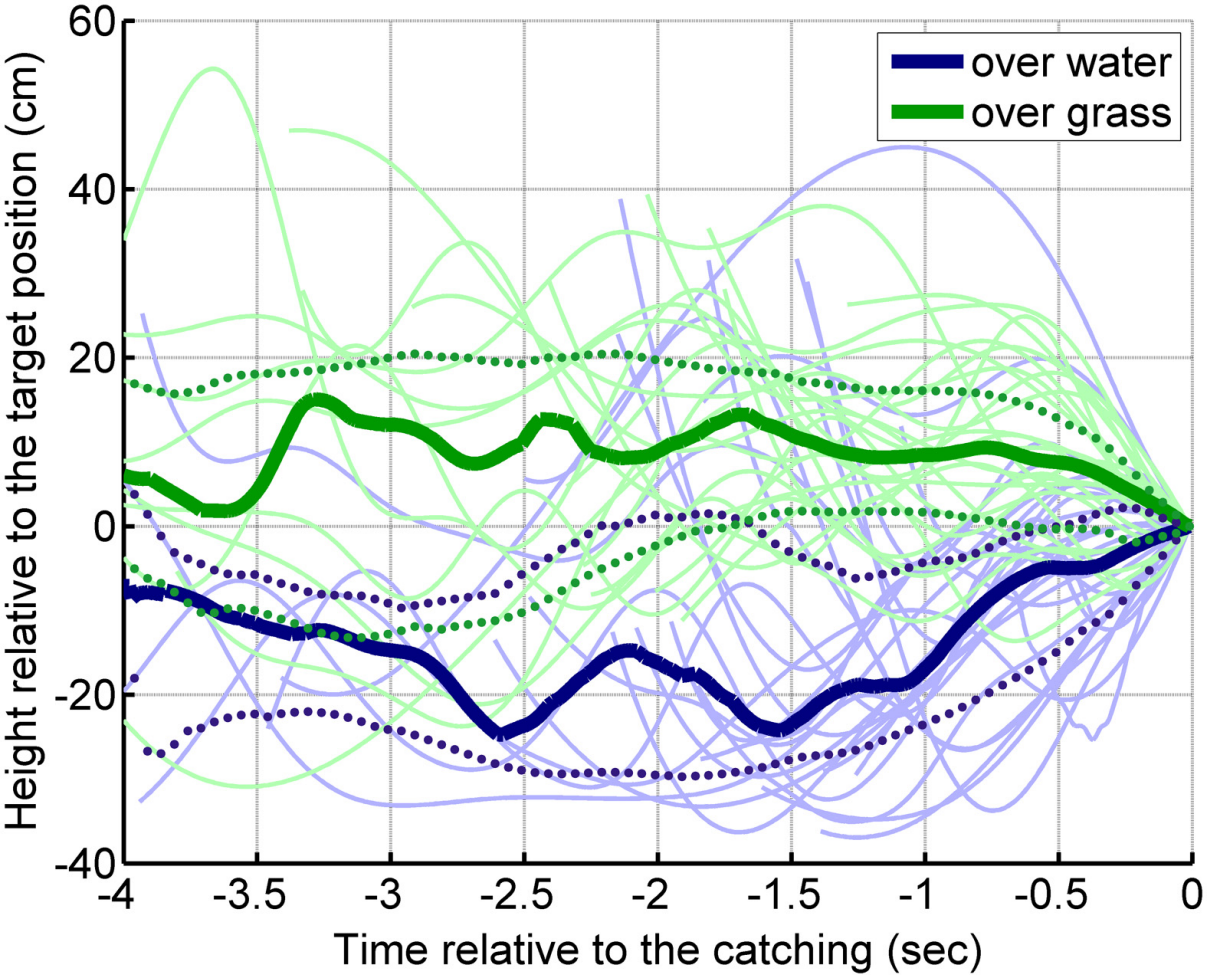
**Flight height of the bats in the last 4 s before making a capture at a target height of 35 cm.** The moment of the capture is shown at 0 s (on the right side of the graph). On average, the bats flew around 10 cm above the target height when they were presented above grass (thick green line). When the targets were presented above water, the bats flew about 10–20 cm below the target height (thick blue line). The strong continuous lines show the median of the flight paths, the dotted lines show the upper and lower quartiles.

The bats' flight paths at the 35 cm target height conditions were reconstructed based on the laboratory video recordings of the last 4 s before capture. The median flight height above the grass surface was about 20 cm higher than above water (Figure 4). The median flight heights show that in the grass surface condition, the bats approached the target slightly from above, whereas in the water condition, the bats approached the target from below. The GLMM showed a significant surface effect [*F*_(1, 47)_ = 48.9, *p* < 0.001], but no individual effect [*F*_(4, 47)_ = 1.26; *p* = 0.30] on flight height.

### Call analysis

**Figure 5:**
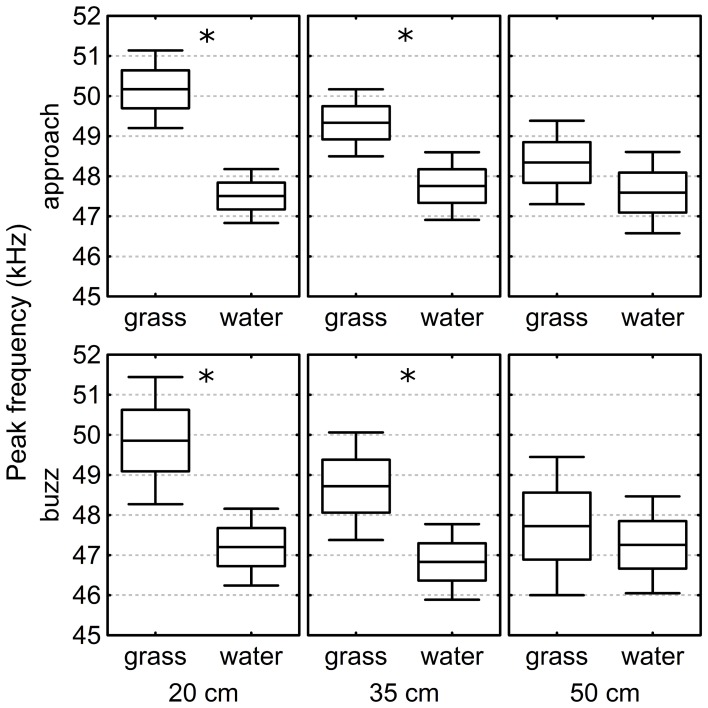
**Peak frequency of the echolocation calls above grass and water in the laboratory.** In both, Buzz and Approach phase, and at target heights of both 20 and 35 cm, the peak frequency was significantly higher above grass than above water. At 50 cm height we found no significant differences in the peak frequency between the two surfaces. The box-plots show the mean, the standard error, and the confidence interval. Stars indicate significant differences (p < 0.001) (see call analysis section).

Two hundred and forty-six echolocation call sequences from Approach phases and 221 sequences from Buzz phases were analyzed in the laboratory recordings. On average, Approach phases contained 13.1 ± 0.6 calls and the Buzz phases contained 9.0 ± 0.34 calls (median ± standard error). The calls' peak frequency was analyzed for Approach and Buzz phase separately. When the targets were presented low above the grass surface, the bats increased the peak frequency of their calls significantly (Figure 5).

The GLMM analysis reveals significant differences in peak frequency between the water and grass surfaces conditions at a target height of 20 cm [GLMM, *F*_(1, 71)_ = 38.5, *p* < 0.001 in Approach and *F*_(1, 65)_ = 12.8; *p* < 0.001 in Buzz phase] and of 35 cm [*F*_(1, 82)_ = 12.2; *p* < 0.001 in Approach phase and *F*_(1, 68)_ = 11.5; *p* = 0.001 in Buzz phase]. No significant differences were found when the targets were 50 cm above the surfaces [*F*_(1, 58)_ = 1.2; *p* = 0.28 in Approach phase and *F*_(1, 53)_ = 0.5; *p* = 0.47 in Buzz phase].

### Ensonification, impulse responses

**Figure 6:**
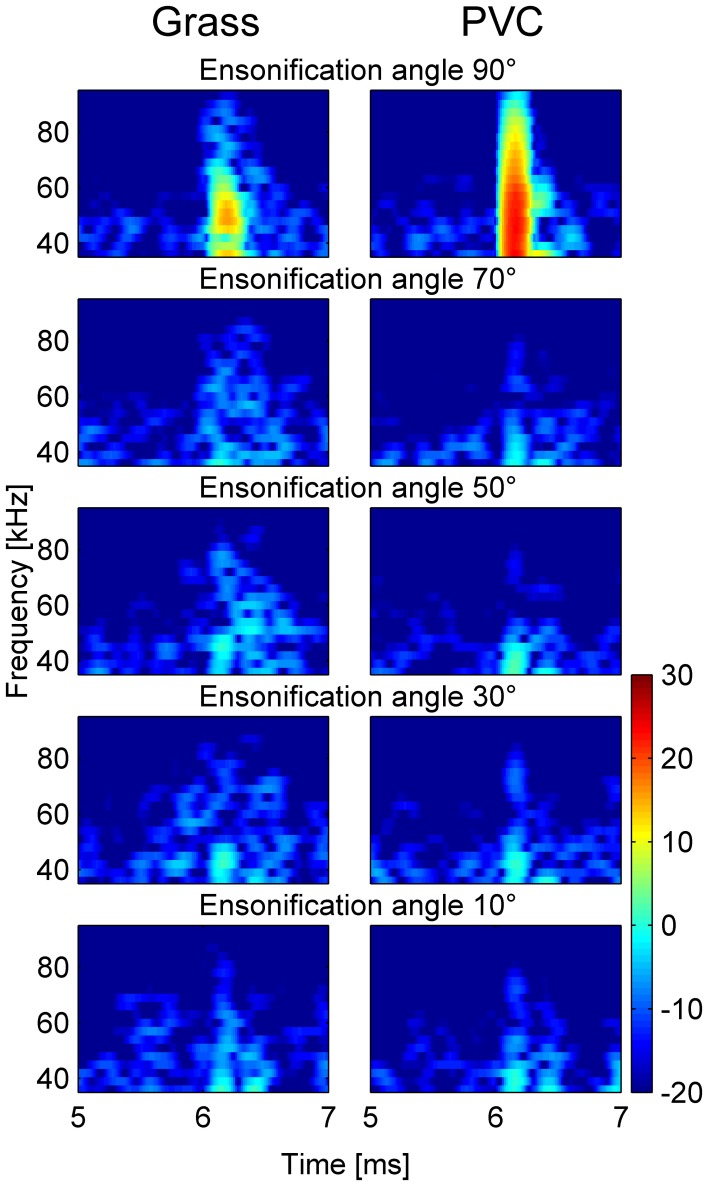
**Spectrograms of the impulse responses of the artificial grass and PVC surface at different ensonification angles.** At an angle of 90° the impulse response of the PVC is stronger and smoother than that of grass (top row). At angles between 30 and 70° (second to fourth row) the impulse responses of the grass surfaces are longer and contain more high frequencies than those of the PVC surface. At 10° there is hardly any difference between the two surfaces.

Two 30 cm discs made of either PVC or artificial grass were ensonified at different angles (Figure 6). At an ensonification angle of 90° (perpendicular ensonification, top row of Figure 6) the IR of the PVC is sharper and louder than that of the grass matting. However, at ensonification angles between 30 and 70°, the IR of PVC is weaker than that of the grass matting, especially at frequencies higher than about 50 kHz. Additionally the IR of the grass matting at these ensonification angles is temporally expanded. At a very small angle (10°) there is hardly any difference between the two surfaces.

## Discussion

In our study we found that for the bat *M. daubentonii*, the detection and discrimination of prey objects decreases at low heights above a clutter surface. This deterioration in psychophysical performance is accompanied by significant increases in both flight height and increases in the peak frequency of the bats' sonar emissions. The good agreement of the data from the Laboratory- and Field experiments corroborates the ecological relevance of the current tasks for the animals in the wild.

In the following we will discuss the data, first with respect to the performance of the bat, i.e., target detection and -discrimination, and second with respect to the behavioral adaptations of the bats, i.e., flight path and echolocation behavior.

### Target detection

In the Laboratory and in the Field experiments, the animals always executed an attack after they had initiated a trial when targets were presented 50 cm above any surface. However, with decreasing target height, the bats attacked less often above the grass surface (Figures 2A–F, green bars) while they still executed attacks above water. The current 2 AFC setup required the bats to find the one thread from which a mealworm is hanging. Unlike in a natural detection task, the general structure of the setup will indicate for the bats where to search for potential prey. Nevertheless, we observed that especially at very low heights above the grass surface, the animals attacked much less frequently.

In an experiment where *M. daubentonii* were trained to catch a mealworm suspended in front of a vertical clutter surface, Siemers and Schnitzler (2004) also found a significant decrease in attacking performance when the target distance was 25 cm or less. Thus, the attacking impairment appears independent of the absolute orientation of the surface (horizontal or vertical).

A particular case of target detection above surface is when the target is on the surface itself. Siemers et al. (2001b) found in their experiment that mealworms which were placed on smooth horizontal linoleum were readily caught, however, when mealworms were placed on the clutter screen, they were almost never caught. Accordingly, Boonman et al. (1998) found that higher duckweed density on natural water surfaces correlates with lower catching success of the bats from the surface. Moreover, bats prefer open waters against waters covered with duckweed. Rydell et al. (1999) also found reduced bat activity above rippled water. Von Frenckell and Barclay (1987) showed that bats' (*M. lucifugus*) foraging activity is higher above calm water than above turbulent water. We have found that attacking performance above the smooth water in the laboratory was the same as above rippled water in the field. These data indicate that although the water in our Field experiment was not smooth, its echo-acoustic reflection properties did not impair the bats' performance. Both literature- and our current findings thus indicate that a clearer definition of clutter is required: the surface tension of a rippled water surfaces acts as a spatial low-pass filter preventing sharp edges on the water surface. Any solid structure protruding from a water surface, however, will produce sharp edges in the surface structure. The artificial grass used in the current experiments consists mainly of such sharp edges. Also the clutter screen used by Siemers and Schnitzler (2004) and the duckweed vegetation of Boonman et al. (1998) included regular sharp edges. Thus, as soon as the background structure includes sharp edges, attacking performance of the bats is dramatically reduced. The question how sensitive the bats' sonar system is to such surface discontinuities has never been formally addressed.

The ensonification experiments showed that the grass surface created stronger echoes, especially at high frequencies, when ensonified at acute angles which represent angles used by bats hunting at low heights above a surface. It is likely that these echoes deteriorate the bats' perception of the three-dimensional shape of the target, and thus lead to the decrease in attacking and discrimination performance with decreasing target height.

Mackey and Barclay (1989) showed that both echo-acoustic clutter and the water-generated noise reduced foraging activity of the bats. By using the artificial grass, we can rule out a detrimental effect of the water-generated noise in our data. Also Siemers and Schnitzler (2004) used a “silent” clutter surface. These data indicate that echo-acoustic clutter introduced by sharp edges is much more likely to limit capture performance for most natural water surfaces.

Schnitzler and Kalko (1998) suggested that prey detection close to a clutter background is determined by the “clutter-overlap zone.” This zone is defined as that prey-clutter distance at which the clutter echo overlaps with the prey echo, and thus inhibits detection. For *M. daubentonii* with a call duration of 1–1.5 ms, the clutter-overlap zone would be around 17–25 cm. Here, we show that detection performance already decreases at a distance of 35 cm to the clutter surface. Thus, a simple distinction in “Detection in the overlap free window” and “No detection in the clutter-overlap zone” is not sufficient to explain the observed hunting performance.

### Target discrimination

In Siemers et al. (2001b) naïve *M. daubentonii* did not spontaneously discriminate between mealworms and dummies (metal and rubber reflectors). The bats had to capture mealworms on a smooth or clutter linoleum screen. They readily captured mealworms on the smooth screen and repeatedly attacked the dummies placed in the same manner. Thus, the following search image was proposed: “small and isolated echo-reflecting objects on or above an acoustically smooth surface.” Our results indicate that when challenged in a two-alternative forced-choice task bats show the ability to discriminate correctly between a mealworm and a similar-sized dummy. However, in nature bats are rarely confronted with such a defined task and it is more often the case that bats have to discriminate between different kinds of objects and prey, e.g., between leaves or little twigs and insects floating on the water surface. Thus, the suggested search image is reasonable, but not generally valid. Boonman et al. (1998) suggested that Daubenton's bats discriminate edible from inedible objects by analyzing changes in the spectrum of subsequent echoes. These changes are evoked by either moving targets, or by the bats moving around the stationary targets, when the targets have aspect-dependent reflection characteristics. In our study, both targets, the mealworm and the dummy, were moving (typically rotating slowly) and thus created changes in the spectrum over subsequent echoes. Yet the bats were still able to discriminate the mealworm from the dummy. Hence, *M. daubentonii* has to have a more sophisticated echo analysis than just analyzing a sequence of echoes which change in their spectral content over time from an echo sequence which is spectrally invariant over time.

### Flight path

Flight paths illustrated in Figure 4 show that above water, the bats fly very close to the surface and attack the prey from below. This behavioral strategy appears to maximize the echo-acoustic enhancement effect (Siemers et al., 2001b, 2005): the lower the height of the bat above the water, the smaller the elevational angle between the direct echo from the prey to the bat and the indirect echo from the prey via the water to the bat. Moreover, when the bat flies close to the water surface, the echo-delay difference between the two echo paths is minimal. As the perceptual echo enhancement will increase with both decreasing angular difference and decreasing temporal difference, the observed flight behavior strongly supports the hypothesis that bats exploit the additional echoes from the water surface to detect and possibly also identify the prey item. As it is true for the aerodynamic ground effect, the increased acoustic impedance of the water surface facilitates the generation of additional prey echoes. Thus, the animals appear to exploit an echo-acoustic ground effect through the spatio-temporal integration of direct echoes from the prey with indirect echoes via water surface. Note, however that this enhancement comes at the expense of misleading spatial cues in the echo, because the indirect echo via the water surface signals the wrong elevation of the prey. To avoid this problem bats could resign to precedence-like auditory strategies, where accurate localization is dominated by the first sound. Precedence effects in the vertical plane have been described in human psychophysics (Litovsky et al., 1997).

Above a clutter surface, the bats flew significantly higher. Increasing the flight height will increase both the angular and temporal differences between the direct echo and the scattered indirect echo (cf. Figure 6) via the clutter surface. Thus, the observed increase in flight height is consistent with the hypothesis that echoes from the clutter surface are not useful for the bat and the bat tries to separate those echoes (both in terms of echo delay and elevational angle) from the direct echoes.

The bats' increased flight height could also be an indication that they fail to properly determine their height above the surface due to the increased and diffuse reflections caused by the clutter surface. As a consequence, they increase the flight height to avoid colliding with the surface as the roughness may indicate a higher likelihood of objects protruding high enough to interfere with the flight path.

Another possible explanation for this adjustment of flight height may lie in echo-acoustic flow-field information. Bhagavatula et al. (2011) showed that, based on visual flow-field information, budgerigars adjusted their flight trajectory always to be closer to that wall which evoked a smaller visual image motion. In our experimental paradigm, the echo-acoustic image motion above artificial grass would be stronger than above water. It is conceivable that such echo-acoustic flow-field information resulted in an adjustment of the flight trajectory to a larger height in the grass condition.

### Echolocation behavior

We analyzed calls from 467 sequences from the Laboratory experiment. Above grass, the bats significantly increased the peak frequency of their echolocation calls with decreasing target height. We stress that these changes in echolocation are small (~3 kHz), but due to the correlation with height and surface, are likely to be a behavioral response of the bat to the surface. Brinklov et al. (2010) showed that *Macrophyllum macrophyllum* increases its peak frequency in a cluttered environment compared to open space. Since the width of the sonar beam is mainly determined by the frequency, these changes in the bats' echolocation calls lead to narrowing of the sonar beam. Suemer et al. (2009) found that *Eptesicus fuscus* tends to increase the second harmonic of its echolocation signals when challenged with a spatial unmasking task. This suggests that a narrow sonar beam is likely to be advantageous when hunting in a cluttered environment, for it reduces the number and intensity of off-axis echoes, and thus increases signal-to-noise ratio.

Due to the downward frequency-modulated structure of the *M. daubentonii* echolocation calls, the increase of call peak frequency is likely to be correlated with decreased call duration. While, due to the strong water reflections picked up by the microphones, an analysis of temporal call parameters appears impossible in our hands, a putative decrease in call duration would further facilitate the temporal separation of prey- and clutter echoes as discussed above (see flight path section).

## Conclusions

The present data provide new behavioral insight into the sophisticated hunting strategies recruited by bats hunting over water. Specifically, the data show that bats not only reliably detect targets above water but can also discriminate targets. When the water surface is covered with a clutter surface (in our case artificial grass, often vegetation in nature), the bats hunting performance, both in terms of detection and discrimination, decreased significantly with decreasing distance to the surface. Also the flight- and ensonification pattern is significantly changed: in contrast to flight over a clutter surface, the bats chose very low flight paths over water which allow for optimal spatio-temporal integration of direct echoes from the prey with indirect echoes via the water surface. This echo-acoustic strategy is analogous to trawling bats exploiting an aerodynamic ground effect (Norberg and Rayner, 1987; Aldridge, 1988; Jones and Rayner, 1991), i.e., the higher impedance of a smooth surface for the lift of an object moving above water. The suggested combination of spatio-temporal integration and precedence-like localization can be viewed as trawling bats not only exploiting an aerodynamic but also an echo-acoustic ground effect.

### Conflict of interest statement

The authors declare that the research was conducted in the absence of any commercial or financial relationships that could be construed as a potential conflict of interest.

## References

[B1] Aldridge H. D. J. N. (1988). Flight kinematics and energetics in the little brown bat, *Myotis lucifugus* (Chiroptera: Vespertilionidae), with reference to the influence of ground effect. J. Zool. 216, 507–517

[B2] Barclay R. M. R. Brigham R. M. (1994). Constraints on optimal foraging: a field test of prey discrimination by echolocating insectivorous bats. Anim. Behav. 48, 1013–1021

[B3] Bates M. E. Simmons J. A. Zorikov T. V. (2011). Bats use echo harmonic structure to distinguish their targets from background clutter. Science 333, 627–630 10.1126/science.1202065 21798949

[B4] Bhagavatula P. S. Claudianos C. Ibbotson M. R. Srinivasan M. V. (2011). Optic flow cues guide flight in birds. Curr. Biol. 21, 1794–1799 10.1016/j.cub.2011.09.009 22036184

[B5] Boonman A. M. Boonman M. Bretschneider F. Van De Grind W. A. (1998). Prey detection in trawling insectivorous bats: duckweed affects hunting behaviour in Daubenton's bat, *Myotis daubentonii*. Behav. Ecol. Sociobiol. 44, 99–107

[B6] Brinklov S. Kalko E. K. Surlykke A. (2010). Dynamic adjustment of biosonar intensity to habitat clutter in the bat *Macrophyllum macrophyllum* (Phyllostomidae). Behav. Ecol. Sociobiol. 64, 1867–1874

[B7] Ciechanowski M. (2002). Community structure and activity of bats (Chiroptera) over different water bodies. Mamm. Biol. 67, 276–285

[B8] Fenton M. B. (1990). The foraging behavior and ecology of animal-eating bats. Can. J. Zool. 68, 411–422

[B9] Hedrick T. L. (2008). Software techniques for two- and three-dimensional kinematic measurements of biological and biomimetic systems. Bioinspir. Biomim. 3:034001 10.1088/1748-3182/3/3/034001 18591738

[B10] Jones G. Rayner J. M. V. (1988). Flight performance, foraging tactics and echolocation in free-living Daubenton's bats *Myotis daubentoni* (Chiroptera: Vespertilionidae). J. Zool. 215, 113–132

[B11] Jones G. Rayner J. M. V. (1991). Flight performance, foraging tactics and echolocation in the trawling insectivorous bat *Myotis adversus* (Chiroptera: Vespertilionidae). J. Zool. 225, 393–412

[B12] Kalko E. K. Schnitzler H. U. (1989). The echolocation and hunting behavior of Daubenton's bat, *Myotis daubentoni*. Behav. Ecol. Sociobiol. 24, 225–238

[B13] Lawrence B. D. Simmons J. A. (1982). Measurements of atmospheric attenuation at ultrasonic frequencies and the significance for echolocation by bats. J. Acoust. Soc. Am. 71, 585–590 7085967 10.1121/1.387529

[B14] Litovsky R. Y. Rakerd B. Yin T. C. T. Hartmann W. M. (1997). Psychophysical and physiological evidence for a precedence effect in the median sagittal plane. J. Neurophysiol. 77, 2223–2226 9114271 10.1152/jn.1997.77.4.2223

[B15] Mackey R. L. Barclay R. M. R. (1989). The influence of physical clutter and noise on the activity of bats over water. Can. J. Zool. 67, 1167–1170

[B16] Norberg U. M. Rayner J. M. V. (1987). Ecological morphology and flight in bats (Mammalia; Chiroptera): wing adaptations, flight performance, foraging strategy and echolocation. Philos. Trans. R. Soc. Lond. B Biol. Sci. 316, 335–427

[B17] Rydell J. Miller L. A. Jensen M. E. (1999). Echolocation constraints of Daubenton's Bat foraging over water. Funct. Ecol. 13, 247–255

[B18] Schnitzler H. U. Kalko E. K. V. (1998). How echolocating bats search and find food, in Bat Biology and Conservation, eds Kunz T. H. Racey P. A. (Washington, DC: Smithsonian Institution Press), 183–196

[B19] Schnitzler H. U. Kalko E. K. V. (2001). Echolocation by insect-eating bats. Bioscience 51, 557–569

[B20] Schnitzler H. U. Moss C. F. Denzinger A. (2003). From spatial orientation to food acquisition in echolocating bats. Trends Ecol. Evol. 18, 386–394

[B21] Siemers B. M. Baur E. Schnitzler H. U. (2005). Acoustic mirror effect increases prey detection distance in trawling bats. Naturwissenschaften 92, 272–276 10.1007/s00114-005-0622-4 15871000

[B22] Siemers B. M. Dietz C. Nill D. Schnitzler H. U. (2001a). *Myotis daubentonii* is able to catch small fish. Acta Chiropterol. 3, 71–75

[B23] Siemers B. M. Stilz P. Schnitzler H. U. (2001b). The acoustic advantage of hunting at low heights above water: behavioural experiments on the European ‘trawling’ bats *Myotis capaccinii, M. dasycneme* and *M. daubentonii*. J. Exp. Biol. 204, 3843–3854 11807102 10.1242/jeb.204.22.3843

[B24] Siemers B. M. Schnitzler H. U. (2004). Echolocation signals reflect niche differentiation in five sympatric congeneric bat species. Nature 429, 657–661 10.1038/nature02547 15190352

[B25] Suemer S. Denzinger A. Schnitzler H. U. (2009). Spatial unmasking in the echolocating Big Brown Bat, *Eptesicus fuscus*. J. Comp. Physiol. A 195, 463–472 10.1007/s00359-009-0424-9 19263055

[B26] Todd V. L. G. Waters D. A. (2007). Strategy-switching in the gaffing bat. J. Zool. 273, 106–113

[B27] Von Frenckell B. Barclay R. M. R. (1987). Bat actibity over calm and turbulent water. Can. J. Zool. 65, 219–222

[B28] Warren R. D. Waters D. A. Altringham J. D. Bullock D. J. (2000). The distribution of Daubenton's bats (*Myotis daubentonii*) and pipistrelle bats (*Pipistrellus pipistrellus*) (Vespertilionidae) in relation to small-scale variation in riverine habitat. Biol. Conserv. 92, 85–91

[B29] Weissenbacher P. Wiegrebe L. (2003). Classification of virtual objects in the echolocating bat, *Megaderma lyra*. Behav. Neurosci. 117, 833–839 12931967 10.1037/0735-7044.117.4.833

[B30] Wund M. A. (2005). Learning and the development of habitat-specific bat echolocation. Anim. Behav. 70, 441–450

[B31] Zahn A. Maier S. (1997). Hunting activity of bats at streams and ponds. Z. Säugetierkd. 62, 1–11

